# Real-World Analysis of the Aging Effects on Visual Field Reliability Indices in Central 10-2 Tests

**DOI:** 10.3390/jpm12101600

**Published:** 2022-09-28

**Authors:** Tomoki Shirakami, Tetsuro Omura, Hiroki Fukuda, Ryo Asaoka, Masaki Tanito

**Affiliations:** 1 Department of Ophthalmology, Shimane University Faculty of Medicine, Izumo 693-8501, Japan; 2 Department of Ophthalmology, Graduate School of Medicine, University of Tokyo, Tokyo 113-8655, Japan; 3 Department of Ophthalmology, Seirei Hamamatsu General Hospital, Shizuoka 430-8558, Japan; 4 Seirei Christopher University, Shizuoka 422-8545, Japan; 5 Nanovision Research Division, Research Institute of Electronics, Shizuoka University, Shizuoka 422-8529, Japan; 6 The Graduate School for the Creation of New Photonics Industries, Shizuoka 431-1202, Japan

**Keywords:** visual field, glaucoma, aging, real-world data, fixation loss, FL, false negative, FN, false positive, FP

## Abstract

We investigated the influence of aging on the reliability indices of visual field (VF) testing using a large dataset of central 10-2 program tests, including 6674 VF tests, which consisted of 1782 eyes of 1094 Japanese subjects (the mean age ± standard deviation was 66.6 ± 14.1 years). All of the combinations for each parameter, except for the pairs between age and fixation losses (FLs) or false positives (FPs) and between pattern standard deviation (PSD) and FPs, had significant correlations (*p* < 0.0001). Among the reliability indices, the false negatives (FNs) had the strongest correlation against age (the correlation coefficient was ρ = 0.21). Each reliability index changes differently with aging. The FLs were the highest in the first 10 s and remained constant after 20 s. The FNs remained constant for 60 s and rose steeply after 70 s. The FPs reached their highest value in 10 s and remained constant after 40 s. In mixed-effect regression analyses in 40-year-old or older subjects, older age was significantly associated with higher FNs (*p* < 0.0001) but not with FLs (*p* = 0.9014) and FPs (*p* = 0.9267). Compared to central 30-2 VF testing, central 10-2 VF tests were associated with smaller FLs (*p* < 0.0001) and FPs (*p* < 0.0001). In central 10-2 testing, age-related deterioration was seen in FNs but not in FLs and FPs. Choosing the 10-2 program over the 30-2 program can be effective in reducing the FL, especially in older cases with severe VF loss. This study highlighted the relationships between age and each reliability index in central 10-2 VF testing.

## 1. Introduction

Glaucoma is the leading cause of irreversible blindness worldwide. The global prevalence of glaucoma in people aged from 40 to 80 years is estimated to be 3.5% [1]. Visual field (VF) loss is detected by semiautomated perimeters, which are widely used to diagnose glaucoma and detect its progression, making them essential for glaucoma management [2]. Static perimetry methods are utilized to estimate retinal function by measuring light sensitivity thresholds. The data of the test results are quantitative, which makes it possible to analyze them statistically. The presence of glaucoma itself and more severe stages of glaucoma are associated with a higher variability of VF testing [3,4,5,6,7]. Other than visual acuity and other visual functions, aging, cognitive function, ethnicity, and mental status can affect the reliability of the VF tests. Therefore, VF testing is intrinsically variable and can have a certain amount of random variability even in healthy, trained, and reliable participants because it is a probabilistic rather than a deterministic examination [8,9,10,11,12,13]. Among the factors, aging is an important factor that affects the results of VF testing. It has been reported that threshold sensitivity declines with aging [14,15]; therefore, the age-related decline in light sensitivity is adjusted by the semiautomated perimeters.

The lower accuracy of the VF test will be reflected by the changes in the reliability indices, which include rates of fixation loss (FL), false negatives (FNs), and false positives (FPs). The evaluation of these indices is critical in monitoring glaucoma progression [2,9,16,17,18,19]. We previously reported the influence of aging on the reliability of VF testing by using a large dataset of a central 30-2 program by the Humphrey Visual Field Analyzer [20]. However, the influence of aging on the reliability indices of VFs tested by the central 10-2 program, a program suitable to test both advanced cases and glaucoma with paracentral scotoma, is not well documented. In this study, we investigated the possible association between age and the VF reliability indices in a large dataset of the central 10-2 program tests to reflect on real-world consequences.

## 2. Materials and Methods

The study adhered to the tenets of the Declaration of Helsinki; the institutional review board (IRB) of Shimane University Hospital reviewed and approved the research (study no. 20080911-1). The IRB approval did not require each patient to provide written informed consent for the publication; instead, the study protocol was posted to the study institutions to notify the participants of the study. To analyze real-world data, all 6674 VF tests using the central 10-2 program, obtained by the Humphrey Visual Field Analyzer (Carl Zeiss Meditec, Dublin, CA, USA) with the SITA Standard, were extracted from the database stored at the Department of Ophthalmology, Shimane University Hospital, between 1988 and 2019. These VF data consisted of 1782 eyes of 1094 Japanese subjects (the mean age ± standard deviation [SD] was 66.6 ± 14.1 years). We also collected the subjects’ age during the VF testing, mean deviation (MD), pattern standard deviation (PSD), and rates of FLs, FNs, and FPs. The possible correlations among each parameter (age, MD, PSD, FL, FN, and FP) were assessed using the Spearman’s rank correlation test. The mean values of each reliability index (FL, FN, and FP) were calculated for each age group stratified by 10 years (i.e., 0–9 years, 10–19 years, and 90–99 years) and were compared between each of those age groups by a one-way analysis of variance followed by the post-hoc Tukey honesty significant difference (HSD) test for the adjustment of multi-pair comparisons. We also performed mixed-effect regression analyses in 40-year-old or older subjects, calculated by age, MD, and PSD as the fixed effects and by subject identification number and tested eye (right or left) as the random effects, in order to assess the influence of each parameter (age, MD, and PSD) on the reliability indices (FLs, FNs, and FPs). All statistical analyses were performed using the JMP Pro statistical software version 15.2.1 (SAS Institute, Inc., Cary, NC, USA).

## 3. Results

**Figure 1:**
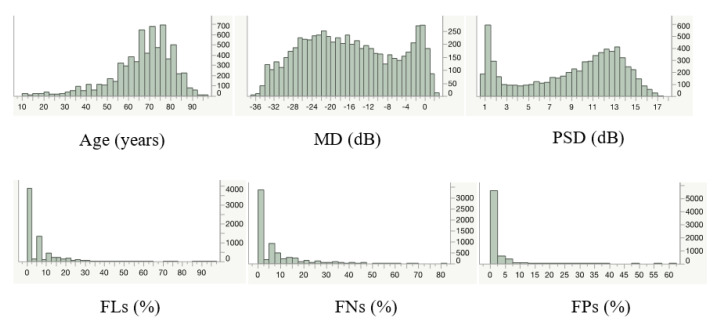
Distribution of the parameters.

Table 1 summarizes the obtained parameters, and Figure 1 shows their distribution. The mean age of the subjects was 66.6 years, with an age range of 0–95 years in this dataset.

The possible associations between age, VF parameters (MD and PSD), and the reliability indices are shown in Table 2. There were no correlations between the pairs of age–FLs, age–FPs, and PSD–FPs. All of the other combinations of parameters had significant correlations (*p* < 0.0001). The strongest correlation against age was with FNs (ρ = 0.21).

**Figure 2:**
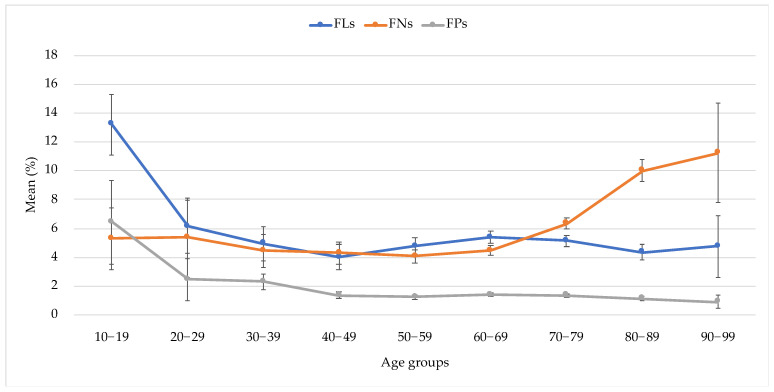
Mean values of FLs, FNs, and FPs in each age-stratified group. FLs, fixation losses; FNs, false negatives; FPs, false positives.

The values of each reliability index in each age-stratified group are shown in Table 3, Table 4 and Table 5 for the FLs, FNs, and FPs, respectively. The FLs, FNs, and FPs were the lowest in their 40 s, 50 s, and 90 s, respectively. Each reliability index changes differently with aging (Figure 2). The FLs were the highest in the first 10 s, decreased at 20 s, and remained constant after 20 s. The FNs remained constant for 60 s and rose up steeply after 70 s. The FPs reached their highest value in 10 s, after which they decreased gradually and remained constant after 40 s.

In order to further assess the influence of aging on the reliability indices, mixed-effect regression analyses on 40-year-old or older subjects were performed (Table 6). Older age was significantly associated with higher FNs (*p* <0.0001), but not with FLs (*p* = 0.9014) and FPs (*p* = 0.9267).

## 4. Discussion

We have previously investigated the relationships between age and the VF reliability indices in a 30-2 dataset [20]. In subjects who are 40 years old or older, the mean ages of the two datasets (the 10-2 test from this study and the 30-2 test from our previous study) were virtually equivalent (68.8 years for the 10-2 and 68.4 years for the 30-2), while the MDs were distinctly different (−16.1 dB for the 10-2 and −7.3 dB for the 30-2) (Table 7). This might reflect the actual operational situation, where the 10-2 perimetry was often tested on people with a relatively poor VF who had difficulty being evaluated with the 30-2 perimetry. For the reliability indices, the mean values for the FLs and FPs were lower in the 10-2 dataset (5.0% and 1.3%, respectively) than in the 30-2 dataset (8.1% and 2.4%, respectively) (*p* < 0.0001 for both comparisons), but the value of the FNs was not different between both datasets (6.0% for the 10-2 and 4.9% for the 30-2; *p* = 0.9364). Since the 10-2 perimetry test requires gazing at a smaller area than the 30-2 perimetry test, it makes sense that the fixation would be better and subsequent false positives would be reduced in the 10-2 test than in the 30-2 test. Especially for the FL, the value seems to have decreased more significantly in the 10-2 test than in the 30-2 test because the same blind spot method is used in the SITA Standard for both the 10-2 and the 30-2 programs [21,22], even though the 10-2 test has a narrower visual field presentation range. The FNs could also be lower in a smaller gazing area, such as in the 10-2 test, but the result was not. This may be due to the fact that the 10-2 subjects had a lower MD value than the 30-2 subjects. It has been reported that the FNs were elevated in eyes with reduced retinal sensitivity, such as those with reduced MD values [3,7,19], and, therefore, it is possible that the improvement of the FN value in the 10-2 program was canceled by the patient’s background of having a lower MD value, although this speculation requires further study. In the central 30 degrees, the variability of the measured threshold values is highly dependent on eccentricity; reproducibility decreases when eccentricity increases [23]. Thus, independent from the MD value, the difference in the test field might explain the differences in the reliability indices between the 10-2 and 30-2 tests observed in this study.

Considering the results of Table 3, Table 4 and Table 5 and Figure 2, we found that the FLs, FNs, and FPs were the lowest in their 40 s, 50 s, and 90 s, respectively. Each reliability index changes differently with aging. The age-related transitions of the FLs and FPs were similar; they were the highest in the first 10 s and remained approximately constant after then. The maximum values of FLs and FPs and the minimum value of FNs might be explained by the influence of high sensitivity and restlessness in the teenage years. The younger age groups likely consisted of more first-time test takers than the older age groups; thus, the learning effects could be another explanation for the relatively stable transition of the reliability indices in the middle-age groups. On the other hand, the steep elevation of FNs in 70 s indicated the aging effect. Interestingly, the same steep elevation of FNs in 70 s was found in our previous study of the 30-2 test [20]. There have been several reports of anatomical and functional aging changes in the macula and perifoveal area [24,25]. The accumulation of debris in the sub-macular Bruch’s membrane increased remarkably at the age of 70 and older [26]. Sixty percent of subjects over the age of 70 exhibited fundoscopic signs of early age-related maculopathy; even those free from these signs demonstrated sensitivity loss [27]. In normal eyes, age-related VF sensitivity loss accelerates after the age of 70 [28]. Thus, these anatomical and functional aging changes occurring around the age of 70 can explain the rapid increase of FNs in the 70 s seen in this study. It is well known that the FNs increase as the MD worsens, and that the MD can be expected to worsen with age as glaucoma severity increases. Thus, the findings in this study contained the bulk effects of aging on the reliability indices.

Regarding the association among the parameters for all ages (Table 2), aging correlated significantly with FNs, with no significant correlation between age and FL or between age and FPs; our previous reports on the 30-2 test showed that all three reliability indices correlated significantly with aging [20]. To exclude the effects derived from the small number of extreme cases, only the subjects aged 40 years or older were included in the multivariate analyses. The mixed-effect regression analyses, with the exclusion of the young subjects, showed a significant correlation between age and FNs, and no correlation between age and FLs or FPs, similar to the univariate analysis. In the previously reported 30-2 study, both the FLs and FNs were correlated with age [20]. The reason for the lack of correlation between FL and aging in the 10-2 test results can be explained by the fact that the mean value and the range of FL values of the 10-2 dataset are smaller than those of the 30-2 dataset, as shown in Table 7. The results suggest that the selection of the 10-2 program rather than the selection of the 30-2 program is an effective way to reduce the FL, especially in older subjects with severe VF loss.

This study has several limitations. First, we did not assess factors such as fatigue and loss of concentration, which might affect the results of the reliability indices. Previous studies reported that a longer testing time was associated with wider variations in the reliability indices [29,30,31,32]. Second, the absence of information regarding ocular pathology and clinical backgrounds, such as visual acuity, was also a limitation of this study. The steep increase in the FNs in the age groups of 70 and older might simply coincide with the occurrence of age-related eye diseases, such as cataract, age-related macular degeneration, and rapidly progressing types of glaucoma, including exfoliation glaucoma and primary angle closure diseases. Accordingly, we cannot exclude the possibility that the age-related worsening of the reliability indices would not be observed if the analyses were performed among subjects without any ocular pathologies and with good physical and phycological health. The results of this study are supposed to reflect real-world outcomes with diverse pathological and background factors. Third, the VF testing used in this study was obtained via several different models of the Humphrey VF analyzer; therefore, the stringency of the reliability index assessment might have changed with consecutive updates. Finally, we discussed the 10-2 results of the current study in comparison with the 30-2 results of the previous study; for this purpose, we only performed simple statistical comparisons of the parameters. In the future, enough statistical comparison between the 10-2 and 30-2 datasets would provide a deeper understanding of the reliability indices.

## 5. Conclusions

This study demonstrates the relationships between age and each reliability index in 10-2 VF tests. FNs were correlated significantly with aging and steeply elevated after 70 s, while the FLs and FPs were relatively constant after 40 s. This age-related change in the FNs could be due to anatomic and functional changes related to aging. Choosing the 10-2 program over the 30-2 program can be effective in reducing the FLs, especially in older cases with severe VF loss.

## Figures and Tables

**Table 1:** Demographic data.

Parameter	Mean ± SD	Range
Age (years)	66.6 ± 14.2	10.0–95.0
MD (dB)	−15.9 ± 10.0	−36.3–2.8
PSD (dB)	9.0 ± 4.6	0.8–17.4
FL (%)	5.0 ± 8.8	0.0–95.2
FN (%)	5.9 ± 9.0	0.0–81.0
FP (%)	1.4 ± 3.1	0.0–62.0

MD, mean deviation; PSD, pattern standard deviation; dB, decibel; FLs, fixation losses; FNs, false negatives; FPs, false positives; SD, standard deviation.

**Table 2:** Possible associations between each parameter tested by a univariate analysis.

ρ * p *	Age (Years)	MD (dB)	PSD (dB)	FL (%)	FN (%)	FP (%)
Age (years)		−0.18	0.07	−0.01	0.21	−0.02
MD (dB)	<0.0001 *		−0.32	0.09	−0.26	0.12
PSD (dB)	<0.0001 *	<0.0001 *		−0.08	0.09	−0.02
FL (%)	0.6158	<0.0001 *	<0.0001 *		0.10	0.30
FN (%)	<0.0001 *	<0.0001 *	<0.0001 *	<0.0001 *		0.10
FP (%)	0.1065	<0.0001 *	0.1803	<0.0001 *	<0.0001 *	

The correlation coefficient (ρ) and the *p* values are calculated by a Spearman’s rank correlation test. * *p* < 0.0001. MD, mean deviation; PSD, pattern standard deviation; dB, decibel; FL, fixation loss; FN, false negative; FP, false positive.

**Table 3:** FL stratified by age.

Age Group (Years)	*n*	Mean (%)	SD	Lower 95%CI	Upper 95%CI	*p*-Value							
						vs. 20–29	vs. 30–39	vs. 40–49	vs. 50–59	vs. 60–69	vs. 70–79	vs. 80–89	vs. 90–99
10–19	67	13.21	1.08	11.10	15.32	<0.0001 *	<0.0001 *	<0.0001 *	<0.0001 *	<0.0001 *	<0.0001 *	<0.0001 *	<0.0001 *
20–29	88	6.14	0.94	4.30	7.98	-	0.9782	0.4866	0.8989	0.9968	0.9813	0.6565	0.9886
30–39	217	4.95	0.60	3.78	6.12	-	-	0.9315	1.0000	0.9992	1.0000	0.9919	1.0000
40–49	383	3.98	0.45	3.09	4.86	-	-	-	0.8678	0.1131	0.2906	0.9989	0.9992
50–59	904	4.77	0.29	4.19	5.34	-	-	-	-	0.7632	0.9781	0.9807	1.0000
60–69	1766	5.37	0.21	4.96	5.78	-	-	-	-	-	0.9965	0.0782	0.9998
70–79	2185	5.14	0.19	4.77	5.51	-	-	-	-	-	-	0.2985	1.0000
80–89	998	4.34	0.28	3.80	4.89	-	-	-	-	-	-	-	1.0000
90–99	66	4.75	1.08	2.63	6.88	-	-	-	-	-	-	-	-

To adjust the multi-pair comparisons, the *p* values are calculated using the Tukey-Kramer’s HSD test between each pair of groups. * *p* < 0.05. SD, standard deviation; CI, confidence interval.

**Table 4:** FN stratified by age.

Age Group (Years)	*n*	Mean (%)	SD	Lower 95%CI	Upper 95%CI	*p*-Value							
						vs. 20–29	vs. 30–39	vs. 40–49	vs. 50–59	vs. 60–69	vs. 70–79	vs. 80–89	vs. 90–99
10–19	63	5.30	8.54	3.15	7.45	1.0000	0.9990	0.9954	0.9763	0.9980	0.9918	0.0014 *	0.0066 *
20–29	86	5.74	12.45	3.07	8.41	-	0.9974	0.9889	0.9488	0.9942	0.9895	0.0004 *	0.0040 *
30–39	213	4.30	7.99	3.22	5.38	-	-	1.0000	0.9998	1.0000	0.0770	<0.0001 *	<0.0001 *
40–49	385	4.97	9.49	4.02	5.92	-	-	-	1.0000	1.0000	0.0013 *	<0.0001 *	<0.0001 *
50–59	820	4.12	7.15	3.63	4.61	-	-	-	-	0.9796	<0.0001 *	<0.0001 *	<0.0001 *
60–69	1695	4.53	7.85	4.16	4.90	-	-	-	-	-	<0.0001 *	<0.0001 *	<0.0001 *
70–79	2051	6.58	9.88	6.15	7.01	-	-	-	-	-	-	<0.0001 *	0.0010 *
80–89	900	10.00	11.47	9.25	10.75	-	-	-	-	-	-	-	0.9815
90–99	58	11.24	13.23	7.76	14.72	-	-	-	-	-	-	-	-

To adjust the multi-pair comparisons, the *p* values are calculated using the Tukey-Kramer’s HSD test between each pair of groups. * *p* < 0.05. SD, standard deviation; CI, confidence interval.

**Table 5:** FP stratified by age.

Age Group (Years)	*n*	Mean (%)	SD	Lower 95%CI	Upper 95%CI	*p*-Value							
						vs. 20–29	vs. 30–39	vs. 40–49	vs. 50–59	vs. 60–69	vs. 70–79	vs. 80–89	vs. 90–99
10–19	67	6.45	11.97	3.53	9.37	<0.0001 *	<0.0001 *	<0.0001 *	<0.0001 *	<0.0001 *	<0.0001 *	<0.0001 *	<0.0001 *
20–29	98	2.57	7.08	1.15	3.99	-	1.0000	0.0548	0.0090 *	0.0385 *	0.0220 *	0.0026 *	0.0465 *
30–39	225	2.24	3.97	1.71	2.76	-	-	0.0057 *	<0.0001 *	0.0008 *	0.0002 *	<0.0001 *	0.0269 *
40–49	399	1.59	4.11	1.19	1.99	-	-	-	0.9990	1.0000	1.0000	0.9449	0.9749
50–59	912	1.22	2.59	1.06	1.39	-	-	-	-	0.9072	0.9876	0.9983	0.9963
60–69	1805	1.40	3.03	1.26	1.54	-	-	-	-	-	0.9998	0.3584	0.9363
70–79	2217	1.33	2.62	1.22	1.44	-	-	-	-	-	-	0.6134	0.9666
80–89	1003	1.15	2.42	1.00	1.30	-	-	-	-	-	-	-	0.9998
90–99	66	0.91	1.97	0.43	1.39	-	-	-	-	-	-	-	-

To adjust the multi-pair comparisons, the *p* values are calculated using the Tukey-Kramer’s HSD test between each pair of groups. * *p* < 0.05. SD, standard deviation; CI, confidence interval.

**Table 6:** Possible association between each reliability index and various parameters in 40-year-old or older subjects by multivariate analyses.

Parameters	FLs (%)			FNs (%)			FPs (%)		
	R	95% CI	*p*-Value	R	95% CI	*p*-Value	R	95% CI	*p*-Value
Age (/years)	0.00	−0.03–0.04	0.9014	0.13	0.09–0.16	<0.0001 **	0.00	−0.01–0.01	0.9267
MD (/dB)	−0.03	−0.06–0.00	0.0503	−0.32	−0.36–−0.28	<0.0001 **	0.03	0.02–0.04	<0.0001 **
PSD (/dB)	−0.18	−0.24–−0.12	<0.0001 **	−0.14	−0.21–−0.06	0.0004 **	0.02	0.00–0.03	0.0401 *

The *p*-values are calculated by mixed-effect regression analyses adjusted by subject identification and tested eye as the random effects. * *p* < 0.05, ** *p* < 0.01. FL, fixation loss; FN, false negative; FP, false positive; R, regression coefficient; CI, confidence interval; MD, mean deviation; dB, decibel; PSD, pattern standard deviation.

**Table 7:** Comparison of the parameters between the VF datasets of the central 10-2 and the 30-2 programs in subjects who are 40 years old or older.

Parameters	Mean ± SD (95% CI Range)		*p*-Value
	10-2 (*n* = 6302)	30-2 (*n* = 32503)	
Age (years)	68.8 ± 10.9 (68.5–69.1)	68.4 ± 11.2 (68.3–68.6)	0.0234 *
MD (dB)	−16.1 ± 9.8 (−16.4–−15.9)	−7.3 ± 8.3 (−7.3–−7.2)	<0.0001 **
PSD (dB)	9.1 ± 4.6 (9.0–9.2)	6.6 ± 4.7 (6.6–6.7)	<0.0001 **
FL (%)	5.0 ± 8.6 (4.7–5.2)	8.1 ± 10.8 (8.0–8.2)	<0.0001 **
FN (%)	6.0 ± 9.0 (5.7–6.2)	4.9 ± 7.1 (4.8–5.0)	0.9364
FP (%)	1.3 ± 2.7 (1.2–1.4)	2.4 ± 4.0 (2.4–2.5)	<0.0001 **

The 30-2 data are adopted from our previous publication [20]. * *p* < 0.05, ** *p* < 0.01, the *p*-values are calculated using the Wilcoxon rank sum test. SD, standard deviation; CI, confidence interval; MD, mean deviation; PSD, pattern standard deviation; dB, decibel; FLs, fixation losses; FNs, false negatives; FPs, false positives.

## Data Availability

Data are fully available upon reasonable request to the corresponding author.
